# Potential Applications of Calcium Pyrophosphate for Bone Regeneration: A Systematic Review

**DOI:** 10.7759/cureus.82983

**Published:** 2025-04-25

**Authors:** Ralitsa Yotsova, Ivaylo Parushev, Stefan Peev, Tsanka Dikova

**Affiliations:** 1 Department of Oral Surgery, Medical University of Varna, Varna, BGR; 2 Department of Periodontology and Dental Implantology, Medical University of Varna, Varna, BGR; 3 Department of Dental Material Science and Prosthetic Dental Medicine, Medical University of Varna, Varna, BGR

**Keywords:** beta-blocker side effects, beta-calcium pyrophosphate, bone graft, bone regeneration, bone scaffold, calcium pyrophosphate

## Abstract

Calcium pyrophosphate (CPP) is a type of calcium phosphate bioceramic and an important intermediate in biological mineralization. It is biocompatible, non-toxic, and osteoconductive, which makes it an attractive material for biomedical applications. Synthetic beta calcium pyrophosphate (β-CPP), used for biomedical applications, is usually obtained by the heat treatment of orthophosphates with a molar ratio of calcium/phosphate (Ca/P)=1. This systematic review aims to investigate the application of CPP for bone regeneration. The research included in vivo studies conducted in this field over the past 50 years. An advanced search was performed using the PubMed, Web of Science, and Scopus databases, and the PRISMA guidelines were followed. Numerous preclinical animal trials have been conducted over the past 50 years to evaluate the use of CPP in the form of granules or porous scaffolds. The study models used were rabbits, dogs, and rats. It was suggested that β-CPP is a biocompatible, absorbable, and osteoconductive material that can be a good alternative to hydroxyapatite (HA) and beta-tricalcium phosphate (β-TCP). The published results are promising but insufficient and do not allow for drawing a definitive conclusion on whether CPP can be used as an alternative to HA and β-TCP in bone regenerative therapy.

## Introduction and background

Innovations in biomedical engineering, combined with rising treatment expectations and needs that traditional methods can no longer meet, are driving rapid advancements in the field of regenerative dental care [1-5]. Today, the convincing results seen with this type of therapy are largely due to the huge variety of biomaterials available on the market, which are being developed to outperform the predecessors [5-10].

Calcium phosphate bioceramics, such as hydroxyapatite (HA) and beta-tricalcium phosphate (β‑TCP), are among the most commonly used biomaterials in bone regenerative therapy [10-13]. HA, as the main part of the inorganic component of bone tissue, is characterized by excellent biocompatibility and osteoconductivity but exhibits a low degree of in vivo resorption [13-15]. On the other hand, β-TCP has good resorbability and biodegradability, making it an attractive biomaterial for rapid bone replacement [16-18]. Usually, combinations of the two ceramics, known as biphasic calcium phosphate ceramics (BCPs), are used in practice. They combine the advantages of both forms [19-21].

Calcium pyrophosphate (CPP) is another representative of calcium phosphate ceramics, which is characterized by a lower calcium/phosphate (Ca/P) ratio (1.0) than that of HA (1.67) and β‑TCP (1.50). This increases the material’s solubility in body fluids and, consequently, its bioresorbability [22,23]. Beta-CPP (β-CPP) also differs from the most common orthophosphates in the structure of its crystal lattice, as it has a tetragonal crystal lattice, while HA and β‑TCP have a hexagonal and rhombohedral lattice, respectively [11,17]. Furthermore, CPP is among the important intermediate products of biological mineralization [24,25].

For biomedical applications, synthetic β-CPP is used, which is usually obtained by heat treatment of orthophosphates with a molar ratio of Ca/P=1. Most often, brushite (CaHPO_4_.2H_2_O) or monetite (CaHPO_4_) are used as precursors [13].

Numerous in vitro studies have found that β-CPP is biocompatible, non-toxic, and osteoconductive, and can be successfully used as a bone substitute material alone or in combination with other biomaterials [26-31].

The lack of toxicity, stability at low pH, and slow wear rate define β-CPP as a promising material for dental applications, e.g., for enamel restoration [32,33]. Furthermore, the drug-loading potential of porous β-pyrophosphate crystals was investigated, demonstrating that they can be successfully applied for local drug delivery during the treatment of periodontitis and peri-implantitis [34].

In vitro studies of macroporous β-CPP scaffolds produced by 3D printing have demonstrated that the material has good biological properties, suggesting its potential use for bone regeneration [35].

In 2011, Lee et al. [36] reported the first clinical trial of β-CPP as a bone graft extender in instrumental posterolateral lumbar fusion. The results showed that the combination of β-CPP with autograft can be used successfully in this procedure and is as effective as autologous bone.

The reported positive results of in vitro studies and the involvement of pyrophosphate ions in the mineralization process suggest that further preclinical studies are necessary to evaluate the use of CPP in bone surgery. With this in mind, this systematic review aimed to investigate the in vivo studies evaluating β-CPP as an alternative to HA and β-TCP in bone regenerative therapy, summarize the current knowledge, and identify the research gaps that necessitate further assessment.

## Review

Materials and Methods

Question

The research question of this systematic review is in accordance with the PCC framework:

P (Patient/Population/Participants) - Animal and human study models for in vivo investigations on CPP-containing biomaterials;

C (Concept) - Application of CPP in bone regeneration; and

C (Context) - Research articles published in the last 50 years (1975-2024)

Research question - Are CPP biomaterials used in bone regenerative therapy (C), and what in vivo studies have been conducted in this area (P) in the last 50 years (C)?

Eligibility Criteria

Inclusion criteria: Preclinical studies, articles presenting in vivo investigations of CPP for bone regenerative therapy, and articles written in English.

Exclusion criteria: Review articles; books; book chapters; abstracts; articles that do not present investigations of CPP in bone regenerative therapy; articles not presenting in vivo experiments; articles in which CPP is present in minimal amounts and/or its impact on bone regeneration is not discussed; articles in languages other than English.

Information Sources

This systematic review followed the guidelines of the Preferred Reporting Items for Systematic Reviews and Meta-Analyses (PRISMA) Statement [37]. It was registered in the Open Science Framework (OSF) registry on 06.01.2025 and can be found at https://osf.io/rnqsj.

Search Strategy

An advanced search was conducted on 10.01.2025 using Web of Science, PubMed and Scopus databases. The search strategy is presented in Table 1.

**Table 1:** Search strategy

Database	Search strategy
Web of Science	ALL=(( ( calcium AND pyrophosphate ) OR ( beta-calcium AND pyrophosphate ) OR ( β-calcium AND pyrophosphate ) ) AND ( ( bone AND regeneration ) OR ( bone AND scaffold ) OR ( bone AND graft ) ))
Scopus	( ( calcium AND pyrophosphate ) OR ( beta-calcium AND pyrophosphate ) OR ( β-calcium AND pyrophosphate ) ) AND ( ( bone AND regeneration ) OR ( bone AND scaffold ) OR ( bone AND graft ) ) AND ( LIMIT-TO ( DOCTYPE , "ar" ) ) AND ( LIMIT-TO ( SUBJAREA , "MEDI" ) OR LIMIT-TO ( SUBJAREA , "DENT" ) ) AND ( LIMIT-TO ( LANGUAGE , "English" ) )
PubMed	( ( calcium pyrophosphate ) OR ( beta-calcium pyrophosphate ) OR ( β-calcium pyrophosphate ) ) AND ( ( bone AND regeneration) OR ( bone AND scaffold ) OR ( bone AND graft ) ) Filters: English, from 1975 - 2025 Sort by: First Author Extended query string: (("calcium pyrophosphate"[MeSH Terms] OR ("calcium"[All Fields] AND "pyrophosphate"[All Fields]) OR "calcium pyrophosphate"[All Fields] OR ("beta-calcium"[All Fields] AND ("diphosphates"[MeSH Terms] OR "diphosphates"[All Fields] OR "pyrophosphates"[All Fields] OR "diphosphoric acid"[Supplementary Concept] OR "diphosphoric acid"[All Fields] OR "pyrophosphate"[All Fields])) OR ("beta-calcium"[All Fields] AND ("diphosphates"[MeSH Terms] OR "diphosphates"[All Fields] OR "pyrophosphates"[All Fields] OR "diphosphoric acid"[Supplementary Concept] OR "diphosphoric acid"[All Fields] OR "pyrophosphate"[All Fields]))) AND ((("bone and bones"[MeSH Terms] OR ("bone"[All Fields] AND "bones"[All Fields]) OR "bone and bones"[All Fields] OR "bone"[All Fields]) AND ("regenerability"[All Fields] OR "regenerable"[All Fields] OR "regenerant"[All Fields] OR "regenerants"[All Fields] OR "regenerate"[All Fields] OR "regenerated"[All Fields] OR "regenerates"[All Fields] OR "regenerating"[All Fields] OR "regeneration"[MeSH Terms] OR "regeneration"[All Fields] OR "regenerations"[All Fields])) OR (("bone and bones"[MeSH Terms] OR ("bone"[All Fields] AND "bones"[All Fields]) OR "bone and bones"[All Fields] OR "bone"[All Fields]) AND ("scaffold"[All Fields] OR "scaffold s"[All Fields] OR "scaffolded"[All Fields] OR "scaffolder"[All Fields] OR "scaffolders"[All Fields] OR "scaffolding"[All Fields] OR "scaffoldings"[All Fields] OR "scaffolds"[All Fields])) OR (("bone and bones"[MeSH Terms] OR ("bone"[All Fields] AND "bones"[All Fields]) OR "bone and bones"[All Fields] OR "bone"[All Fields]) AND ("graft s"[All Fields] OR "grafted"[All Fields] OR "graftings"[All Fields] OR "transplantation"[MeSH Subheading] OR "transplantation"[All Fields] OR "grafting"[All Fields] OR "transplantation"[MeSH Terms] OR "grafts"[All Fields] OR "transplants"[MeSH Terms] OR "transplants"[All Fields] OR "graft"[All Fields])))) AND ((english[Filter]) AND (1975:2025[pdat]))

Study Selection, Data Collection, and Data Items

The primary publication data required for this study (title, abstract, authors, year of publication, journal, and DOI) were exported to an MS Excel (Microsoft Corp., Redmond, WA, US) spreadsheet. Duplicate records were removed, and the abstracts were subjected to the eligibility criteria independently by two reviewers (R.Y. and I.P.). The full texts of the remaining articles were then assessed for eligibility, and those that did not meet the inclusion criteria were removed. Data extracted from each article meeting the eligibility criteria included authors and year of publication, composition of the investigated biomaterial, study design, implantation period, control material or other experimental groups, and methods for assessment. Cohen‘s kappa coefficient (κ) was used for measuring the inter-rater agreement level.

Risk of Bias Assessment

The risk of bias (ROB) was assessed independently by two reviewers, R.Y. and I.P., using the SYstematic Review Centre for Laboratory animal Experimentation (SYRCLE) ROB tool for animal studies [38]. The Risk-Of-Bias VISualization (ROBVIS) tool [39] was applied to visualize the results.

Results

Study Selection

A total of 670 potential articles were identified in the initial search of the three databases. In the next stage, duplicates were removed, and the number of articles was reduced to 638. After evaluating their abstracts, 43 potential articles remained for inclusion in the review (with an almost perfect level of agreement κ=0.976; 95% CI 0.942-1.000). Their full texts were reviewed in detail, and reports not meeting the eligibility criteria were excluded. Finally, seven studies were included in the present systematic review. The inter-rater level of agreement was strong; κ=0.78 (95% CI 0.549-1.000).

Study Exclusion

Thirty-six articles were excluded from the review for the following reasons: reports not discussing CPP (5), reports not discussing bone regeneration (19), studies not presenting in vivo experiments (8), and studies in which CPP was observed in insufficient amounts (4).

**Figure 1:**
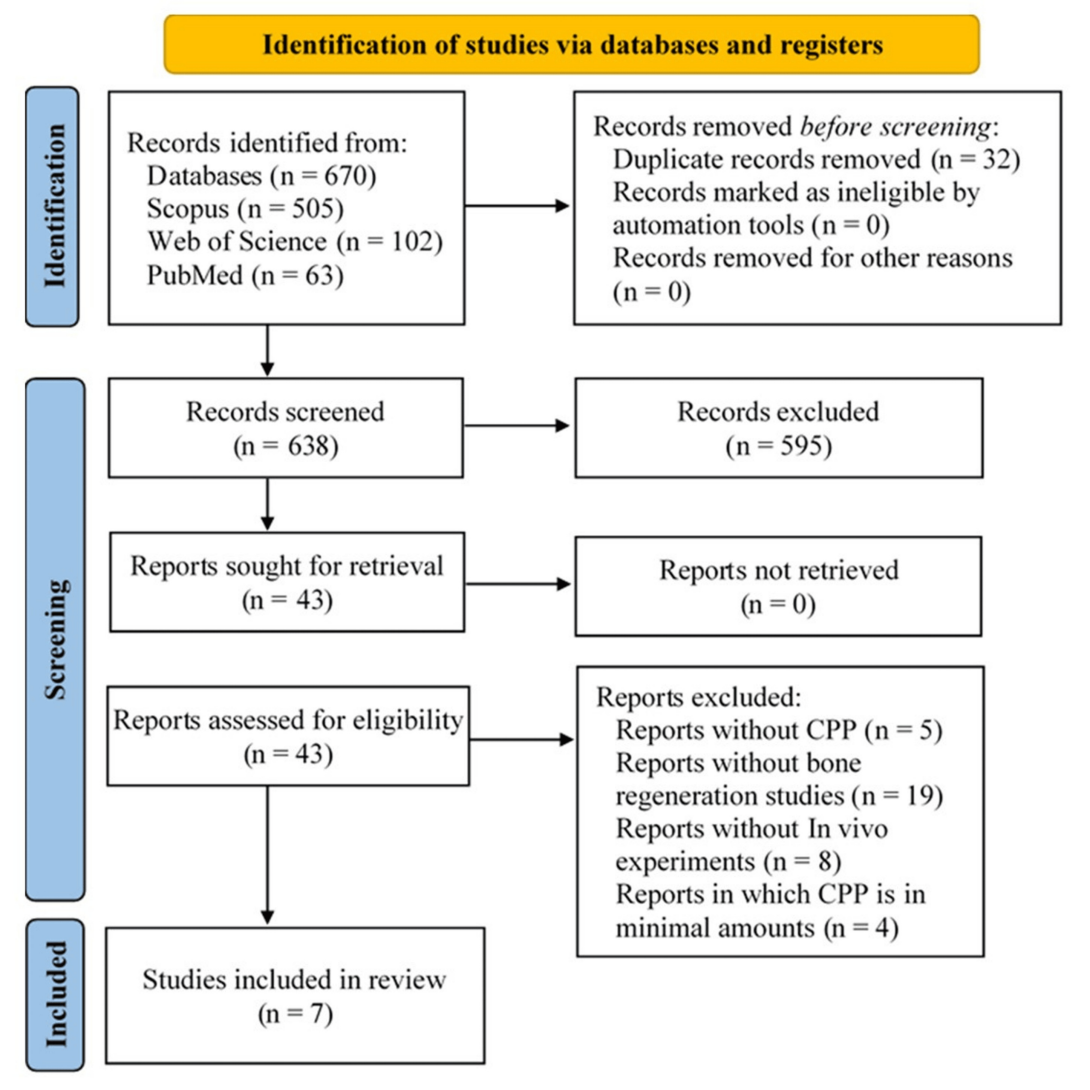
PRISMA flow chart of this review

Figure 1 illustrates a PRISMA flow chart of the selection process.

The characteristics of the included studies are presented in Table 2.

**Table 2:** Details of the articles included in the present review β‑CPP, beta calcium pyrophosphate; β-TCP, beta tricalcium phosphate; BCP, Biphasic calcium phosphate; CPP, Calcium pyrophosphate; HA, Hydroxyapatite; RhBMP-2, Recombinant human bone morphogenetic protein-2; SEM, Scanning electron microscopy

Author, Year	Composition of biomaterial	Study model	Implantation period	Control material/ other experimental groups	Methods for assessment
Dias et al., 2006 [40]	Glass ceramics granules containing β-CPP	Rabbit	2, 4 and 12 weeks	β‑TCP	SEM; Histology
Koo et al., 2006 [41]	CPP granules	Rabbit	8 weeks	None	Histology; Histomorphometry
Lee et al., 2003 [42]	β‑CPP, porous implants	Dog	8 and 20 weeks	HA	Radiographic; Histology
Lee et al., 2015 [43]	β‑CPP, sponge-type porous scaffolds	Rabbit	12 weeks	HA	Histology; Histomorphometry
Lin et al., 1995 [44]	Sintered β‑CPP with Na_4_P_2_0_7_.1OH_2_O addition; disc-shaped porous blocks	Rabbit	1, 2, 4, 6, 8 and 12 weeks	empty defective cavity without bone substitute inside	Histology; Histomorphometry
Naga et al., 2014 [45]	CPP-coated porous alumina scaffolds	Rat	5 months	none	SEM; Histology
Park et al., 2016 [46]	CPP granules; CPP/RhBMP disks	Rat	4 and 8 weeks	BCP; BCP/RhBMP	Histology; Histomorphometry

Quality Assessment

**Figure 2:**
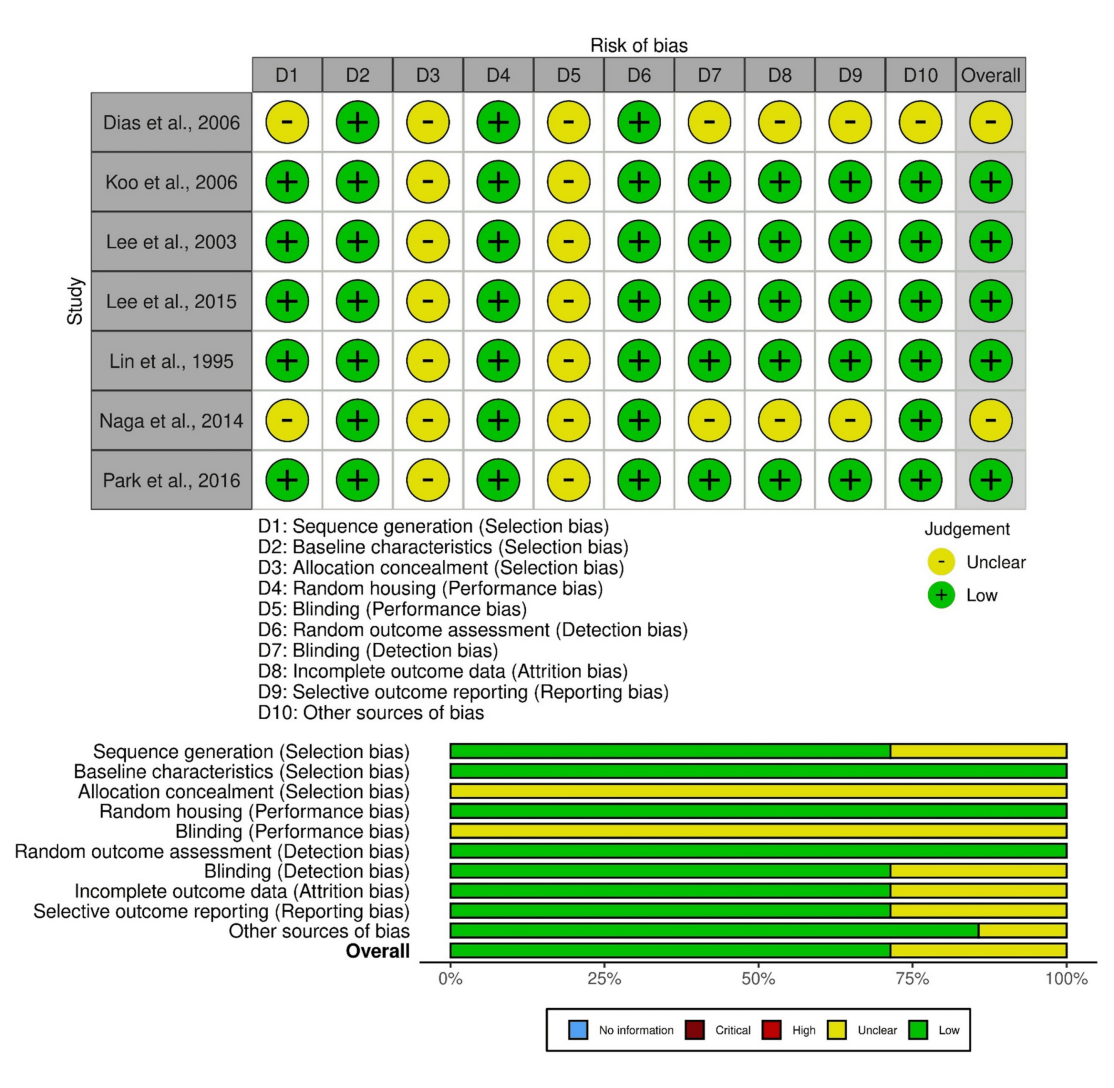
Risk of bias assessment References: [41-46]

According to the quality assessment, five studies presented a low ROB [41-44, 46] and two studies presented a medium ROB [40, 45], allowing for their inclusion in further evaluation (Figure 2).

Study Characteristics

The studies included in this systematic review present in vivo studies of CPP in the form of granules [40,41,46], porous scaffolds [42-44], or coatings on alumina scaffolds [45]. Four studies used a rabbit model [40,41,43,44], one study used a dog model [42], and the remaining two used a rat model [45,46]. All the included articles used histological studies to evaluate the results. The study periods ranged from one to 20 weeks. The control groups were established bone substitute biomaterials in four of the studies, namely β-TCP [40], HA [42,43], and BCP [46]. In all of them, CPP demonstrated similar results to the materials in the control groups.

Discussion

Biological mineralization is a complex process in which HA and other inorganic calcium phosphates are formed. One of the important intermediates in this process is CPP. The formation of pyrophosphate ions (P_2_O_7_^4-^) prevents pathological mineralization during bone formation, as they act as inhibitors of calcification [24,25,47,48].

Besides being a natural intermediate in biological mineralization, β-CPP possesses some important qualities of bone regenerative materials, such as biocompatibility, absorbability, and osteoconductivity. However, additional preclinical studies are necessary to allow its application in clinical practice. Therefore, various in vivo studies have been conducted over the years.

Studies in a rabbit model have found that biodegradable glass-ceramic granules containing 73% β‑CPP exhibit good osteoconductive properties. The experimental granules were obtained by controlled crystallization and sintering. The study involved 24 Japanese white male rabbits whose tibias were implanted with the materials for two, four, and 12 weeks. The authors concluded that the studied granules are a good alternative to HA and β-TCP and can be applied as bone grafts in clinical studies [40].

The osteoconductive activity of β-CPP in the form of granules, produced by a sintering process, has also been confirmed in a study observing their application as a bone substitute material around 16 titanium implants in rabbit tibia [41].

The potential applications of porous β-CPP scaffolds as bone substitutes have been investigated. The experimental specimens are characterized by interconnected pores with a size of 300-500 μm and a total porosity of 75%, and are fabricated by the polyurethane template method and sintering. The results of a study in a canine bone defect model demonstrated that the porous β-CPP framework initially acted as an osteoconductive scaffold and then underwent a remodeling process [42]. Later, Lee et al. conducted comparative histological studies in 20 New Zealand white rabbits and reported that porous β-CPP implants showed more desirable characteristics for application as a bone graft substitute compared to porous HA implants. This can be explained, on the one hand, by the satisfactory osteoconductive properties of β-CPP and, on the other, by the faster resorption rates of these scaffolds and the possibility of them being completely replaced by new bone [43].

In 1995, Lin et al. investigated the behavior of porous blocks of sintered β-CPP doped with 5% Na_4_P_2_0_7_.1OH_2_O and implanted in a rabbit tibia [44]. The histological examinations demonstrated that extracellular dissolution of the scaffolds occurred initially, followed by grinding and migration of the particles, and replacement of the synthetic material with newly-formed bone.

The biocompatibility and osteoconductivity of β-CPP have attracted the attention of researchers for its application as a coating for porous scaffolds. For example, Naga et al. investigated scaffolds made of inert ceramic alumina and coated with CPP by impregnating the porous bodies in brushite, and undergoing subsequent heat treatment [45]. In this way, the poor biological properties of aluminum blocks and the unsatisfactory mechanical properties of phosphate ceramics are simultaneously compensated. The results of in vitro and in vivo studies suggested that these scaffolds can be used as bone substitutes or bone fillers.

It has been established that β-CPP bone grafts can be successfully used as carriers of bone morphogenetic proteins, e.g., recombinant human bone morphogenetic protein-2 (rhBMP-2). The results of some in vivo experiments in 50 Sprague-Dawley rats suggested that the treatment of β-CPP with rhBMP-2 significantly improved bone regeneration [46].

The small number of in vivo trials (only seven for the last 50 years), with the last article being published in 2016, and the lack of clinical trials are the major limitations of this systematic review. Future studies should focus on these limitations and provide more preclinical data, potentially allowing further clinical trials.

## Conclusions

CPP has been evaluated as a potential biomaterial for bone regenerative therapy due to its biocompatibility, absorbability, and osteoconductivity. Additionally, pyrophosphate ions play a very important role in the mineralization process. Some published in vitro and in vivo studies in this area have shown promising results. However, the current evidence is limited and not sufficient to draw a definitive conclusion on whether β-CPP can be used as a viable alternative to HA and β-TCP. To confirm or reject these findings, further preclinical and clinical studies are required.
